# Design and Implementation of a New Real-Time Frequency Sensor Used as Hardware Countermeasure

**DOI:** 10.3390/s130911709

**Published:** 2013-09-04

**Authors:** Raúl Jiménez-Naharro, Juan Antonio Gómez-Galán, Manuel Sánchez-Raya, Fernando Gómez-Bravo, Manuel Pedro-Carrasco

**Affiliations:** Department of Electronic Engineering, Computers, and Automatic, University of Huelva, Ctra Huelva-La Rábida, s/n, 21819 Huelva, Spain; E-Mails: jgalan@diesia.uhu.es (J.G.); msraya@diesia.uhu.es (M.S.); fernando.gomez@diesia.uhu.es (F.G.); mpedro@diesia.uhu.es (M.P.)

**Keywords:** hardware security, VLSI, clock glitching, reverse engineering, frequency sensor

## Abstract

A new digital countermeasure against attacks related to the clock frequency is –presented. This countermeasure, known as frequency sensor, consists of a local oscillator, a transition detector, a measurement element and an output block. The countermeasure has been designed using a full-custom technique implemented in an Application-Specific Integrated Circuit (ASIC), and the implementation has been verified and characterized with an integrated design using a 0.35 μm standard Complementary Metal Oxide Semiconductor (CMOS) technology (Very Large Scale Implementation—VLSI implementation). The proposed solution is configurable in resolution time and allowed range of period, achieving a minimum resolution time of only 1.91 ns and an initialization time of 5.84 ns. The proposed VLSI implementation shows better results than other solutions, such as digital ones based on semi-custom techniques and analog ones based on band pass filters, all design parameters considered. Finally, a counter has been used to verify the good performance of the countermeasure in avoiding the success of an attack.

## 1. Introduction

Traditionally, attacks have been produced in the communication channels between the emitter and receptor, or taking advantage of weaknesses in the mathematics of algorithms. The behavior and/or the structure of the hardware can also provide important information about the system, but the black box model avoids this possibility. However, recent advances in electronics have achieved the replacement of the black box model with gray or white box models in which hardware details are opened [1–3]. These details can be used by an attacker to obtain private information about the system. An example of attack exploiting the gray box model is reverse engineering [4,5]. In this case, the attacker only needs access to the channels of the main signals in order to monitor them, and to determine the behavior of the system. An example of an attack exploiting the white box model is the microprobing technique [6,7]. In this case, the attacker can monitor the whole structure of the hardware.

An important issue is the intention of the attack that can be classified in three groups [8]: obtaining secret information, copying the behavior and/or structure of the system, and provoking malfunctions in the system. An example of attacks related to obtaining secret information is the clock glitching [9,10], where glitches are injected in the clock line at certain times (for example, the check of an encryption loop), modifying the system behavior and increasing the vulnerability. The attacker can obtain secret information by comparing the output of both a correct and an attacked behavior of the system. Clock glitching attacks are among the more critical ones due to the fact the system owner can ignore the attack has happened. A typical attack to obtain information about the system behavior/structure is reverse engineering [4]. In this case, all signal values of the system are monitored by the attacker obtaining its behavior through the information flow. This attack usually needs to decrease the frequency of the clock line in order to obtain the necessary time to monitor the system signals. Concerning attacks related to malfunction of the system or a denial of service [11], a very simple way to achieve it is to increase the frequency of the clock line. In this case, the system does not have the time necessary to perform its operations, and thus, its behavior is become unpredictable causing a malfunction.

According to the above comments, the alteration of the clock signal frequency can be used to increase the vulnerability of the system in many ways. This situation is only possible when the clock line is accessible by the attacker, or it must be introduced externally. The use of external clocks is not unusual in devices with private information. For example, smartcards need an external clock set to a certain frequency [12,13]. Therefore, this work focuses on the attacks that modify the frequency of the clock signal.

The main issue to design a countermeasure is that no attacks can be performed between the countermeasure and the system to be protected. This detail involves two design considerations. Firstly, both the countermeasure and the protected system must be implemented in the same security region. Secondly, both the countermeasure and the protected system must always operate simultaneously, that is, the countermeasure must not have specific inputs and/or supply signals that an attacker can use to stop its operation.

The paper is organized as follows: the behavior and architecture of the proposed frequency sensor is described in Section 2. In Section 3, the sensor is characterized by simulation. In Section 4, the proposed implementation is compared with the same architecture using a semi-custom technique and an analog band pass filter. Section 5 verifies the suitability of the frequency sensor in a case of study based on a counter. Finally, some conclusions are drawn in Section 6.

### 1.1. Description of the Proposed Frequency Sensor

**Figure 1.**
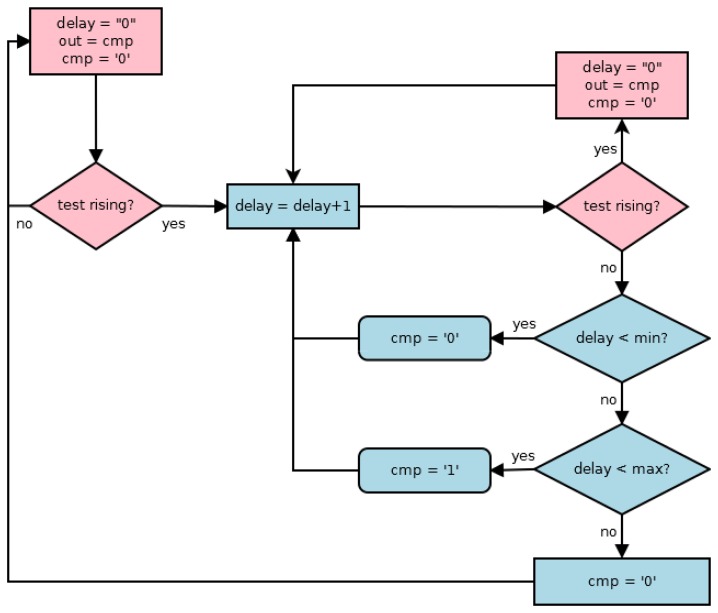
Behavior of the sensor operation.

The main aim of the frequency sensor is to determine when the period of the *clock* signal is within an allowed range counting the cycles number of a timing scale during a *clock* signal cycle. Figure 1 shows the flowchart of the behavior of the proposed sensor. The initialization of the sensor includes the initialization of both measured (henceforth *delay*) and *cmp* signals, while the *out* signal stores the previous value of the *cmp* signal, *i.e.*, the result of the early cycle in *clock* (*test* from now on) signal. When a new active transition (a rising transition has been considered) arrives in the *test* signal, the *delay* signal increases until another new rising transition arrives or its maximum value has been achieved. During a same cycle of the *test* signal, the behavior of the *cmp* signal is the following: when the *delay* signal does not achieve the minimum value, the *cmp* signal is low, indicating the period is out of the allowed range; when the *delay* signal is between its minimum and maximum values, the *cmp* signal is high, indicating the period is within the allowed range; finally, when the *delay* signal is higher than the maximum value, it is low indicating the period is out of the allowed range again. A new initialization of the sensor begins with a new rising transition in the *test* signal, and so, the *cmp* signal is stored in *out* signal. At this moment, the frequency sensor obtains two data points: if the period is within the allowed range, and, if so, the period of the *test* signal stored in the *delay* signal. These data are used by the output block in order to implement the final action of the sensor.

### 1.2. Architecture of the Sensor

**Figure 2.**
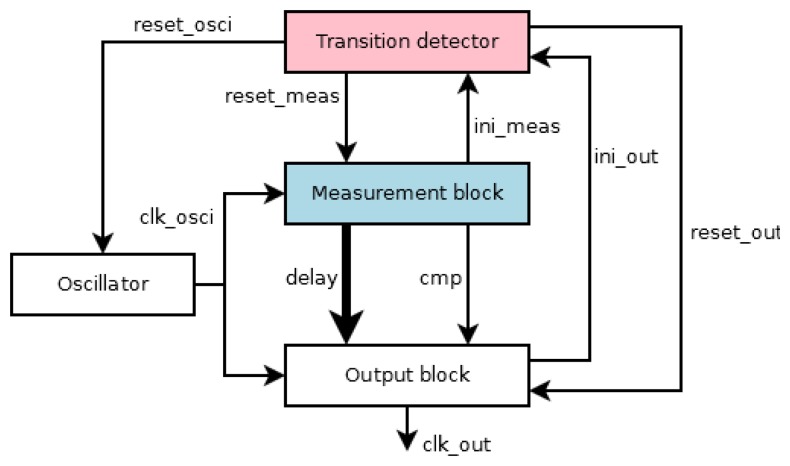
Architecture of the sensor at block level.

Figure 2 shows the building blocks of the proposed sensor fulfilling the above behavior. The local oscillator provides a known time scale that is free of attacks, whereas the aim of the transition detector is to initialize the operation of the sensor. The measurement block indicates an attack situation depending on the period of the *test* signal. These three components form the core of the sensor and provide information related to the existence of an attack and the period of the *test* signal. Finally, the output block provides the final action of the sensor. A typical action is to advise the protected system to erase some confidential information. However, in this work, a free attack clock is generated, and thus, the operation of the system can continue despite any attempted attack. A free attack clock has the same period of the *test* signal when not under attack, or a flat level otherwise.

#### 1.2.1. Implementation into a VLSI ASIC

A typical platform to implement systems with confidential information is an ASIC. Thus the countermeasures used to protect it should be implemented in the same security region, *i.e.*, into the same ASIC. Therefore, this work is focused on a VLSI implementation of the proposed behavior.

Though the main secure systems, such as smart cards, usually use an operation cycle of a few MHz [13], a full-custom technique is used in the implementation of the frequency sensor in order to obtain high performances in terms of resolution and allowed range of periods. Moreover, a well-established 0.35 μm standard CMOS technology and the use of the static CMOS logic family avoid the verification process from being influenced by technological parameters.

This implementation is configurable in the main timing parameters of the sensor, that is, the timing resolution and the allowed range of periods. The first parameter (the minimum timing step that the sensor can discriminate) is configured in the local oscillator because this resolution is the period of the operation cycle in the sensor. The range is configured in the measurement block and, depending on the final action, it influences in the implementation of the output block. Following, the operations and implementations of each block are described in detail.

#### 1.2.2. Local Oscillator

The main difficulty is the lack of a clock signal to perform timing measurements, because any external signal can be manipulated. Therefore, the generation of an attack-free known timing scale must be local. This timing scale is generated using a local oscillator, and thus, the period of the *test* signal can be measured. Many oscillator implementations have been reported in literature [14–16]. In this work, the local oscillator has been implemented according to the following considerations: firstly, the architecture of the protected system must not be altered, and hence, this oscillator does not replace the global oscillator, so the fan-out requirements are not very stringent. Secondly, the occupied area of the sensor must not limit the size of the protected system. A possible implementation can be a feedback chain of inverters.

**Figure 3.**
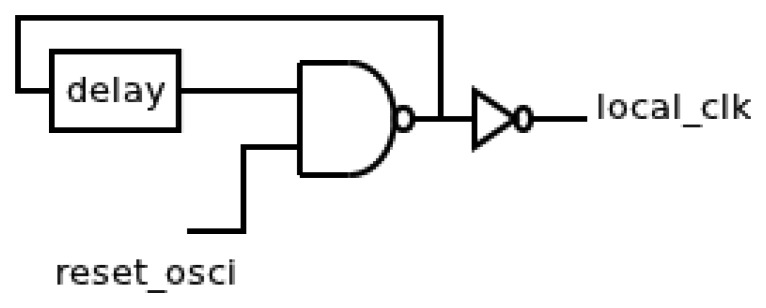
Implementation of the local oscillator.

Figure 3 shows the generic implementation of the oscillator that consists of a typical digital solution based on a feedback chain of inverters. Thus, the period of the oscillator is controlled by the delay of the feedback chain. This delay is determined by their number and sizes. A Negated AND (NAND) gate has been also added to include the reset functionality. Finally, an output inverter allows isolating the oscillator from the rest of the sensor. Therefore, the period of the oscillator will not be influenced by changes in the rest of the sensor due to any possible variations in its configuration.

The operation of this oscillator is as follows: when a rising transition arrives in the *test* signal, the local clock (*clk_osci* signal from now) must be stopped until the other components are initiated. After that, the oscillator restarts until a new active transition happens. This behavior needs an asynchronous reset signal (*reset_osci* signal from now) in the oscillator. The aim of this signal is twofold. Firstly, it avoids timing violations in memory elements during the initialization of the rest of components. Secondly, it guarantees a same timing offset between a new active transition in *test* signal and the first cycle in the *clk_osci* signal.

#### 1.2.3. Transition Detector

The aim of this component is to provide the initialization signals of the other components when a new active transition arrives in the *test* signal. The input of this component is the *test* signal that is the source of the attack. Then, it is the most critical component of the sensor because it must avoid that possible attack spreads to the rest of the sensor.

**Figure 4.**
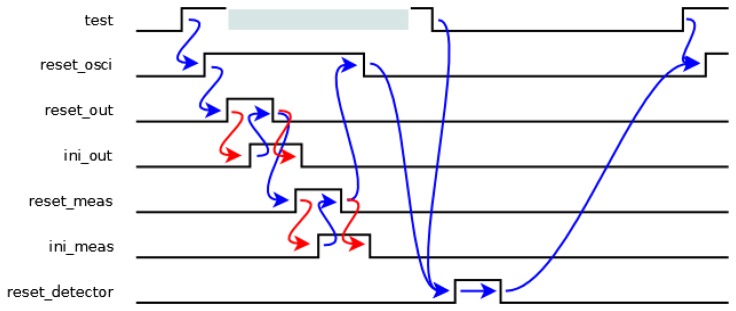
Behavior of the transition detector.

The initialization cycle of the sensor is shown in Figure 4, where the blue transitions are generated by the transition detector, while the red transitions are generated by other components. When a rising transition arrives in the *test* signal, the oscillator must be stopped until the initialization of the rest of the components has finished, avoiding timing violations in memory elements. Then, the *reset_osci* signal is activated. Firstly, the output block is initiated since it uses data from the measurement block. Therefore, the *reset_out* signal is activated until its initialization has finished indicated by an *ini_out* signal. Secondly, the measurement block undergoes the same process with the *reset_meas* and *ini_meas* signals. Next, when all the components have been initiated, the *reset_osci* signal is deactivated, and hence, the oscillator begins to oscillate again. Finally, a new detection process can begin when both the initialization process has finished (*reset_osci* signal is low again) and the *test* signal is low. This situation is indicated by a pulse in the *reset_detector* signal.

The implementation of the transition detector has two main considerations. Firstly, it is an asynchronous implementation, owing to the fact the local oscillator must be stopped during its operation. In fact, the operation shown in Figure 4 corresponds to a speed independent model [17], because both the *ini_out* and *ini_meas* signals ensure the correct behavior though the initialization times are unbounded. Secondly, the minimum value of the allowed period depends on the delay of this component, and thus the delay must be as low as possible. This fact can be achieved considering the minimum number of changes of signals.

**Figure 5.**
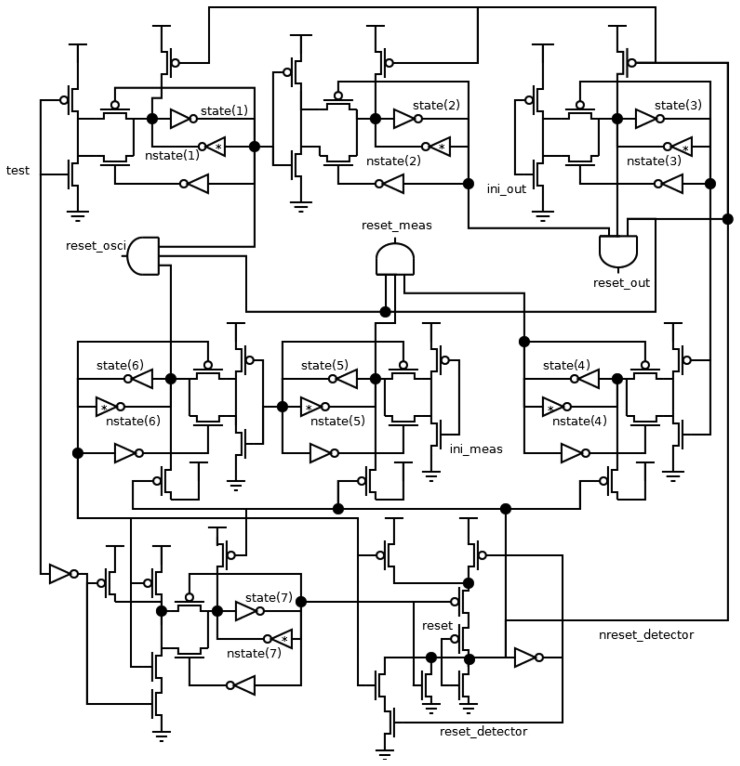
Schematic of the transition detector.

The schematic shown in Figure 5 for the transition detector can guarantee both mentioned considerations. S*tate* signals are activated sequentially when a rising transition arrives in the *test* signal from the *state(1)* signal until the *state(7)* one (the activation of the last signal involves a double condition: the activation of the *state(6)* one and the deactivation of the *test* one). Therefore, each *state* signal is stored in a latch made up of two feedback inverters and a CMOS switch. One of these inverters, labeled with an asterisk, is more resistive, and thus, a possible information collision is avoided. Finally, the control signal of the CMOS switch must be generated after data is stored in the latch. The timing offset between two consecutive *state* signals can be implemented by a delay element (made up by the latch) or an unbounded wait (from both the *ini_out* and *ini_meas* signals). In the case of a delay, it is necessary to ensure that the transitions in both signals are not simultaneous. In the case of a wait, the transition detector must wait until other components finish their initialization. Finally, when the *reset_detector* signal is activated, all *state* signals are deactivated. The values of *state* signals indicate a time span in Figure 4, and hence, all reset signals depend on the values of these signals, *i.e.*, the *reset_out*, *reset_meas* and *reset_osci* signals are activated during the time span between the *state(2)* and *state(3)*, *state(4)* and *state(5)*, and *state(1)* and *state(6)* signals, respectively. Also, the *reset_detector* signal must not be active.

#### 1.2.4. Measurement Block

The aim of this block is to indicate the existence of an attack. Its main components are a counter and a comparator. The counter measures the period of the *test* signal using the local oscillator as timing scale. The comparator decides if the period is within the allowed range, and it stores this decision during the initialization of the sensor.

**Figure 6.**
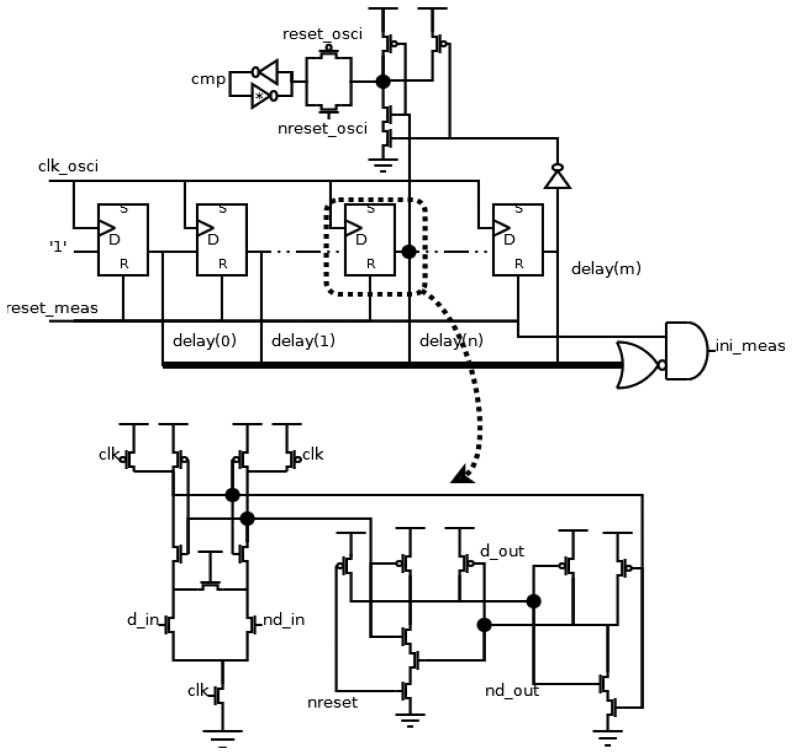
Implementation of the measurement block.

The scheme of this block is shown in Figure 6. The counter has been implemented using a shift register, resulting in an easy implementation of the comparator. With this selection, the limits of the allowed range of periods are shown in Equation (1), where the values of *n* and *m* to a certain allowed range can be derived. The information about the period of *test* signal is also stored in *delay* signals, and it is used later in the output block:

(1) $$\begin{array}{c} \text{min limit}=\text{initialization time}+\text{local period}\times n\\ \text{max limit}=\text{initialization time}+\text{local period}\times m \end{array}$$

The flip-flops in the shift register are implemented using a SAFF (Sense-Amplifier-based Flip-Flop) [18]. This configuration shows a good behavior in the main design parameters [19]. Specifically, it achieves a low switching noise and a high robustness due to the use of a differential topology, and hence, it is a good selection in this application field. The architecture of a SAFF configuration consists of a differential structure, generating the timing behavior, and a RS latch, filtering the pre-charge value. In our case, the asynchronous reset operation has been implemented in the RS latch.

The comparator consists of the AND operation between both the *delay(n)* and *not delay(m)* signals during the operation time (*reset_osci* signal is low), and the decision is indicated in the *cmp* signal. During the initialization time (*reset_osci* signal is high), the *cmp* signal is stored asynchronously, using a similar latch to those used in the transition detector. Finally, it is necessary to generate an *ini_meas* signal. It is activated when all *delay* signals are at low level during the block initialization (*reset_meas* is at high level).

#### 1.2.5. Output Block

**Figure 7.**
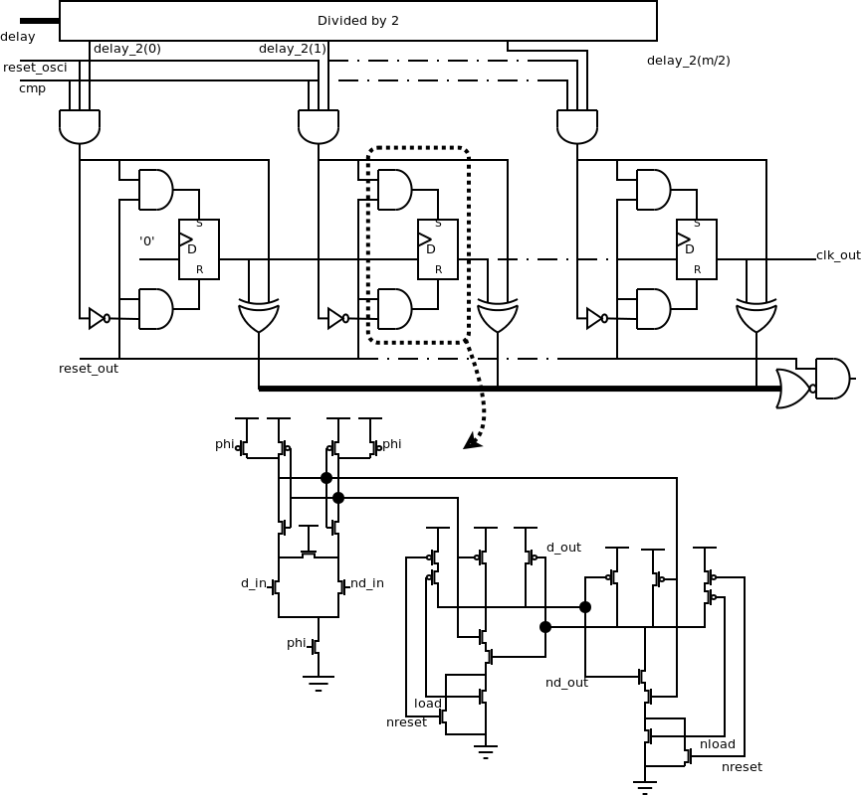
Implementation of the output block.

This block generates the output of the frequency sensor. The designer must only change this block if a different output is desirable. The other blocks, *i.e.* the local oscillator, the transition detector and the measurement block, are always the same. In this case, the output of the sensor is a attack-free clock signal, and has the same period as the *test* signal when it is within the allowed range, or a flat level when it is outside the allowed range. The implementation of the output block is shown in Figure 7.

The timing information about the period of the *test* signal (*delay* signals from the measurement block) is divided by 2 in order to obtain the information about the semi-period. Since the counter used is a shift register, the “divisor by 2” element passes the odd elements of the *delay* signals. When the period of the *test* signal is within the allowed range (both *cmp* and *reset_osci* signals are active simultaneously), this value is used to asynchronously initialize the output block, activating reset or set signals of each flip-flop. Otherwise, the initialized value will be zero, due to the fact only reset signals of flip-flops are activated. The information is stored in a shift register, and it is taken out in each local cycle. Therefore, the *clk_out* signal is only at high level during a semi-period of the *test* signal when its period is within the allowed range. Finally, an *ini_out* signal must be generated during the initialization of the block (*reset_out* signal is high) and the stored values are equal to the initialization ones. A SAFF configuration has been also used to implement the flip-flops of this block. Again, the asynchronous load is implemented in the RS latch as shown in Figure 7.

### 2. Results

The proposed implementation has been verified and characterized by simulation at the transistor level. The simulator used is Eldo Simulator from Mentor Graphics (Meudon, France). The simulation environment fixes the allowed period to 10 ns, *i.e.*, a frequency of 100 MHz, and thus, the characteristics of the sensor are set. Specifically, the local oscillator uses two inverters in the delay path, that is, the minimum number of inverters, and hence, the sensor is configured to the maximum timing resolution. With this resolution, the allowed period of 10 ns is achieved with *n* and *m* parameters equals to 2 and 3, respectively, in the measurement block. Finally, the *m* parameter sets the components of the output block.

**Figure 8.**
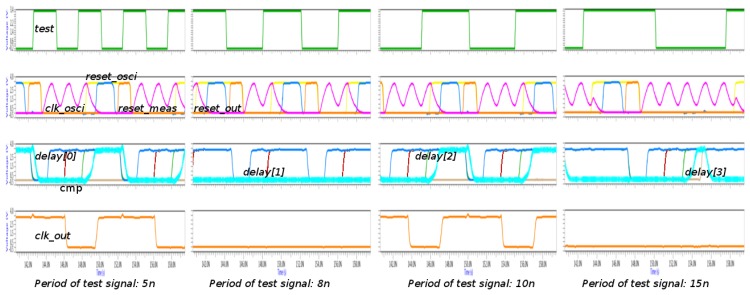
Behavior of VLSI sensor varying the period of *test* signal.

A study of the proposed implementation varying the frequency in the *test* signal is shown in Figure 8. Four groups of waveforms are shown: the *test* signal is on the top; the main signals of oscillator (*clk_osci*) and transition detector (*reset_osci*, *reset_meas* and *reset_out*), together with the main signals of the measurement block (*cmp* and *delays*) are in the middle of the figure; and finally, the output of the sensor is on the bottom. It is interesting to note that the *clk_osci* signal is so quick that it does not reach 0 V. This issue is not important due to the fact the voltage is below the limit of low level.

Four different cases are shown: the period in the *test* signal is lower than the initialization time (5 ns case); the period is higher than the initialization time, but lower than the allowed period (8 ns case); the period is within the allowed period (10 ns case); and the period is higher than the allowed period (15 ns case). In all cases, it is observed that the initialization cycles are completed: while the *reset_osci* signal is active, the *clk_osci* signal is flat and the *reset_out* and *reset_meas* signals are activated sequentially. After the deactivation of the *reset_meas* signal, the *reset_osci* signal is also deactivated, and the *clk_osci* signal begins to oscillate again. The decision is adopted depending on the *delay* signals: *cmp* signal is active when the *delay(2)* and *delay(3)* signals are high and low, respectively. The period is within the allowed range when the *cmp* signal is active during the following initialization cycle, such as the 10 ns case. In this case, the *clk_out* signal has the same period as the *test* signal. The 5 ns case is special since all cycles in the *test* signal are not considered due to the fact its period is shirter than the initialization time. However, the situation is not problematic because the *clk_out* signal has a period within the allowed range. This fact is due to the delay between two considered cycles is within the range.

Using the above configuration, the timing characteristics of the sensor are: the initialization time is 5.84 ± 0.6 ns, the sensor precision is 1.91 ± 0.3 ns, the lower allowed limit is 9 ns, the higher allowed limit is 10 ns, and the sensor resolution is 2 ns. These characteristics have been obtained using a step of 1 ns in the variation of the period of the *test* signal.

Following, the area characterization is considered. An estimation of the area occupied is included in Table 1. In this study, the *num_delay* parameter is the number of inverters in the feedback chain in the local oscillator, and the *m* parameter determines the maximum limit of the allowed range.

**Figure 9.**
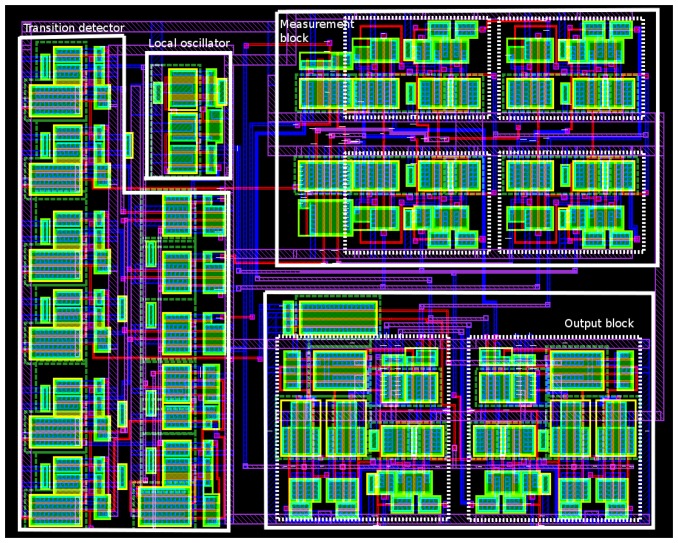
Layout of VLSI sensor with *num_delay* and *m* parameters equals to 2 and 3 respectively.

The estimation has been made considering the number of transistors and their size (width and length product). Using a 0.35 μm standard CMOS technology, the used sizes (W/L) have been 1.4 μm/0.4 μm and 5.6 μm/0.4 μm for NMOS (N-channel MOS) and PMOS (P-channel MOS) transistors respectively; and the sizes of weak transistors (in the delay path) have been 1.4 μm/1.4 μm and 5.6 μm/1.4 μm for NMOS and PMOS transistors, respectively. Obviously, the above sizes have been scaled by the number of transistors connected serially. The number of transistors is not a limiting factor considering the average number of transistors in a micro-controller, which is a typical component in a security system. As the area of connections is not considered in this study, a layout of the configuration used in simulations has been performed, shown in Figure 9. The four components of the sensor can be distinguished. The layout has a size of 16,649.28 μm^2^; therefore, the correction factor including the area of connections may be estimated to be about 23.

It can be noted that the resolution time ranges from 1.44 ns (better case: 3.6 V and 5 °C) to 4.79 ns (worse case: 1.5 V and 50 °C), and the initialization time from 5.09 ns to 15.02 ns. The sensor exhibits a suitable behavior when the supply voltage is higher than the sum of the threshold voltages of NMOS and PMOS transistors, typical constraint of standard CMOS logic family. Specifically, the performance of the frequency sensor is right from 1.5 V at low and typical temperatures, and from 1.8 V at high ones. As the sensor behavior shows a low dependence on the environment, the performance deviations in the frequency sensor are expected to be similar to deviations of the protected system. Therefore, the behavior of the whole system should be still correct.

**Figure 10.**
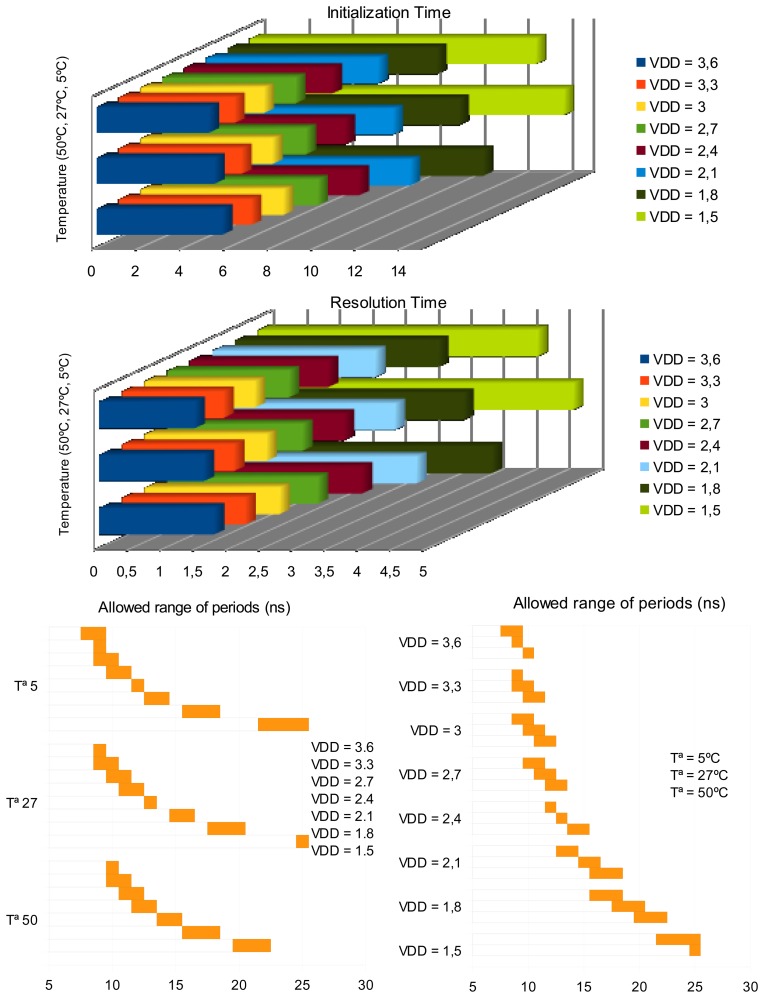
Study of the sensor behavior varying temperature and supply voltage.

Finally, the study of the frequency sensor is completed considering the effects on the main parameters, *i.e.* initialization time, resolution time and the allowed range of periods changing the behavioral conditions, particularly supply voltage and temperature. This issue is important due to the fact that an attacker can use the variations in the environment conditions in order to increase the vulnerability of the system. Figure 10 shows the results obtained using the same configuration of the sensor. The variation of the supply voltage has been considered from 0.9 V (because it is similar to the threshold voltage of a transistor) to 3.6 V (because it is higher than the nominal value, 3.3 V.) with a step of 0.3 V. Concerning the variation of temperature, the chosen values are low (5 °C), typical (27 °C) and high (50 °C). The timing dependence of the sensor is the same than for any integrated digital circuit, *i.e.*, the timing parameters increase when the supply voltage decreases, and the expected influence with the temperature causes that the timing parameters increase at high temperatures.

## 3. Comparison

### 3.1. FPGA Solution

The same architecture has been implemented in a FPGA (Field Programmable Gate Arrays) device verifying its proper behavior with a semi-custom technique based on standard cells [20]. Table 2 shows a comparison between FPGA and VLSI implementations of the sensor considering the main timing parameters, which are the initialization time and sensor resolution. In the case of the resolution, the highest resolution has been included, and thus the local oscillator uses the minimum number of elements in both implementations. From this comparison, the influence of the technique (full-custom or semi-custom) and the platform (VLSI or FPGA device) of implementation is to halve the timing parameters. Also, it is interesting to note that a frequency of 100 MHz could not be into the allowed range using an implementation based on FPGA device, because the initialization time is higher than 10 ns.

### 3.2. Analog Solution

The traditional implementation of a frequency sensor is an analog circuit based on a band-pass filter. The output of the filter provides the same frequency than the clock input when it is within the allowed range. Otherwise, the output must be flat. However, this filter must meet specific characteristics to avoid that specific terminals can be used by an attacker in order to increase the vulnerability of the whole system. It is interesting to note that the functionality to advise the rest of the system is not implemented in an analog implementation, and hence, the defense is to stop to the system when attacked.

A difference with typical analog filters is that the limits of power supply voltages must be the same than the ones of the digital parts, that is, V_DD_ and ground. This situation is due to the fact the sensor must not have specific signals that an external attacker can use in order to isolate it from the rest of the system. Therefore, when the input frequency is out of range, the filter provides low output swings around to V_DD_/2. This level corresponds to the unknown logic value in the digital part of the global system.

**Figure 11.**
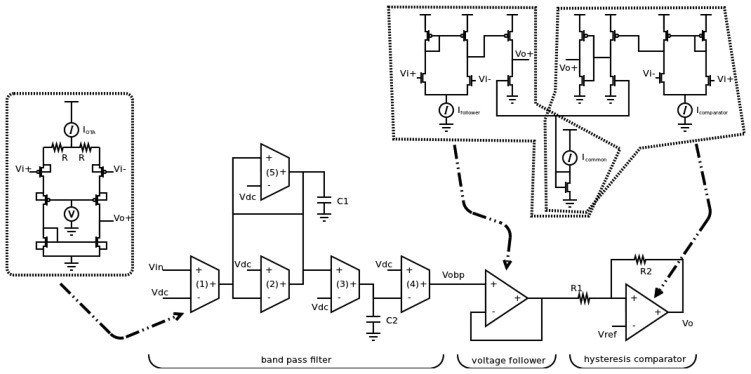
Scheme to block level of the analog sensor.

A scheme at the block level of a band-pass filter used as the analog sensor is shown in Figure 11. The analog sensor consists of a band-pass filter, obtaining the timing behavior, a hysteresis comparator, avoiding the unknown logic value, and a voltage follower, reducing the coupling. The main constraint of the filter for this application is the frequency selectivity which is directly related to the quality factor, and hence, to the resolution time of the sensor. A transconductance-C (gm-C) filter [21,22] has been used as it can operate at higher frequencies due to its open-loop topology as regards to its counterpart active RC filter, where the closed-loop operation limits the bandwidth. The transfer function of the second-order biquad Gm-C filter, the centre frequency w_0_ and the quality factor Q are given by Equations (2–4), respectively:

(2) $$\frac{V_{BP}}{V}=\frac{s\cdot \frac{g_{m1}}{C_{1}}}{s2+s\cdot \frac{g_{m2}-g_{m5}}{C_{1}}+\frac{g_{m3}\cdot g_{m4}}{C_{1}\cdot C_{2}}}$$

(3) $$w_{0}=\sqrt{\frac{g_{m3}\cdot g_{m4}}{C_{1}\cdot C_{2}}}$$

(4) $$Q=\frac{C_{1}}{g_{m2}-g_{m5}}\cdot w_{0}$$

This topology allows a practical design fixing all transconductances at the same value (except for g_m5_) as well as capacitors C_1_ and C_2_. A large frequency selectivity (large Q) can be achieved adjusting the transconductor g_m5_, which is configured as a partial positive feedback as shown in Equation (4).

When the frequency of input signal is out of the pass band, the output signal presents small swings around V_DD_/2. This variation can change some logic values and the effect of the sensor could be canceled. Therefore, it is necessary the use of a hysteresis comparator to avoid that these variations reach the sensor output. The use of the comparator involves the use of a voltage follower to minimize the coupling effect between the filter and the comparator.

**Figure 12.**
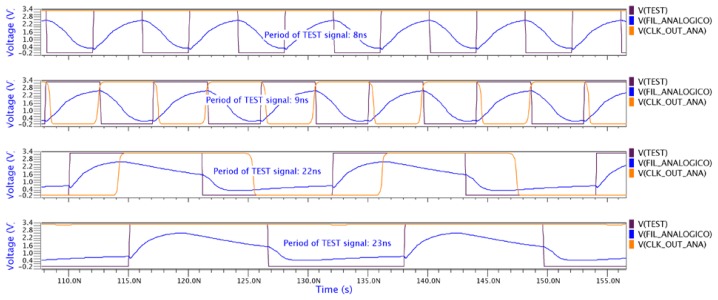
Timing behavior of the analog sensor.

The bandwidth has been measured varying the frequency of *test* signal and observing the behavior of the *clk_out* signal (Figure 12). The *test* signal is not filtered when the *clk_out* signal is not flat. From this study, the allowed range of period is between 9 ns and 22 ns. In Figure 12, it can be appreciated that the *clk_out_ana* signal is saturated. This situation is expected because the input data in the filter (the *test* signal) varies between both saturation levels.

The comparison of results is shown in Table 3. The main design parameters have been included in the comparison: power consumption (considering *test* signal frequencies within and out of the allowed range), timing resolution (considered as the minimum allowed range), the delay between both the *test* and *clk_out* signals, and the area occupied. The estimation of this area has been calculated by the sum of each transistor channel area (the product of both length and width of transistor), while the areas of passive elements and sources are not considered. We can appreciate that the proposed implementation in this paper exhibits better behavior than the analog implementation in all parameters.

## 4. Study Case and Discussion

The system used for the verification of the VLSI frequency sensor consists of a simple system based on a counter. A similar element, the program counter of a micro-controller, is attacked when an attacker wants to obtain the secret key in a DES algorithm [9,23]. Therefore, the authors believe that the use of a counter is significant enough to verify the defense of the sensor, and its low complexity aids to the verification process.

**Figure 13.**
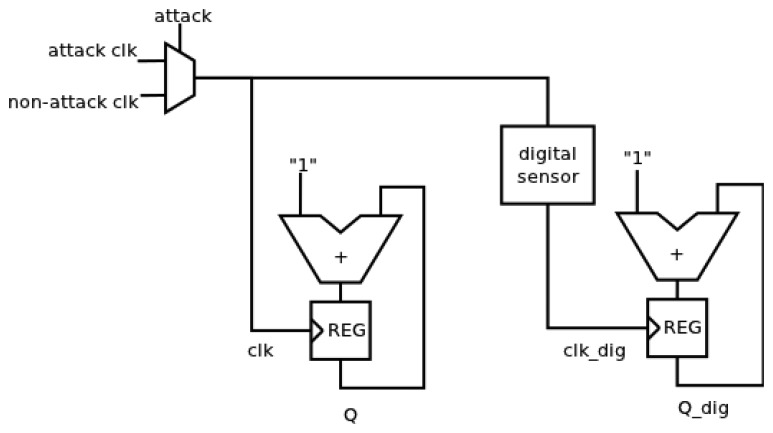
Scheme to block level of the verification environment corresponding to the proposed VLSI sensor.

The simulated system is shown in Figure 13. The attacked system consists of an adder (using a ripple carry configuration) and a 12 bits register. Two systems have been considered: one of them has no countermeasure and the other one has used the proposed sensor as a countermeasure. An intelligent attack has been configured, that is, when the most critical operation (all bits in the adder must change) must be performed, the frequency is changed to the attack frequency. The attack block is modeled with a multiplexer.

**Figure 14.**
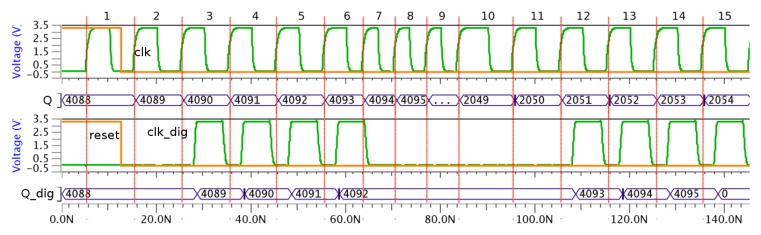
Behavior of an attacked accumulator without and with the proposed VLSI sensor.

The behavior of this attack is shown in Figure 14. When there are no countermeasures, the attack is successful because the sequence of the accumulator is not correct, that is, it is not from 4,095 to 0 but 4,095 to 2,048, and hence, all bits are not generated correctly.

However, when the countermeasure is included, the attack is not successful due to the fact the sequence of the accumulator is correct. In this case, the sequence is from 4,095 to 0. Also, no frequency increase is observed in the *clk_dig* signal; only that it is flat when this change is produced in the *clk* signal. It is interesting to note that both the *Q* and *Q_dig* signal values are different because the number of operation cycles is also different. This difference is due to the influence of the *reset* signal on the sensor behavior.

## 5. Conclusions

Attacks related to the clock signal, such as clock glitching or reverse engineering, are among the most critical attacks because no evidence is left. Therefore, the ignorance of the attack means that the attacker can use the information longer. All these attacks are based on modifying the frequency of the clock signal, either locally in the case of clock glitching or globally in the case of reverse engineering. Conceptually, the typical countermeasure against this kind of attacks is a band-pass filter.

A digital implementation of a frequency sensor has been presented, and this sensor has been used as a countermeasure. The proposed solution is configurable in resolution time and allowed range, and has been designed in order to enlarge the number of applications where it can be used. The implementation has been verified with an integrated design using a 0.35 μm standard CMOS technology (based on a full-custom technique). The main characteristics are an initialization time of 5.84 ns, a minimum resolution time of 1.91 ns and a low occupied area (16,649.28 μm^2^ in the case of the layout of Figure 9).

The proposed implementation has been compared with other schemes used as countermeasures, such as an analog implementation based on a band-pass filter. The proposed sensor exhibits a better behavior in all design parameters considered: initialization time, resolution time, power consumption and area occupied. The influence of supply voltage and temperature in the proposed sensor has been also studied, showing that the sensor behavior is not especially sensitive to these two parameters.

Finally, the solution has been verified in a real attack case. The chosen attack has been a clock glitching. A counter has been used as demonstrator because it is very similar to the program counter, that is a typical objective in systems based on micro-controllers. The simulation results show that when the countermeasure is included, the success of the attack is avoided.

## Figures and Tables

**Table 1.** Estimation of hardware resources depending on the configuration of the VLSI sensor.

**Components**	**N Transistors**	**Estimated Area (μm^2^)**	**Configuration: Ttors/area (μm^2^)**
Local oscillator	12 + 2 × num_delay	15.12 + 9.8 × num_delay	16/34.72
Detector transition	173	276.64	173/276.64
Measurement block	44 + 38 × (*m* + 1)	50.12 + 36.4 × (*m* + 1)	196/195.72
Output block	12 + 84 × *floor*[(*m* + 1)/2]	21.84 + 95.76 × *floor*[(*m* + 1)/2]	180/213.36
Total	241 + 2 × num_delay + 38 × *m* + 84 × *floor*[(*m* + 1)/2]	363.72 + 9.8 × num_delay + 36.4 × *m* + 95.76 × *floor*[(*m* + 1)/2]	563/720.40
Total (including the correction factor)		8365.56 + 225.4 × num_delay + 837.2 × *m* + 2202.48 × *floor*[(*m* + 1)/2]	563/16569.2

**Table 2.** Comparison between FPGA and VLSI implementations, considering timing parameters.

**Timing Parameters**	**FPGA Implementation(Standard Cell)**	**VLSI Implementation(Full-Custom)**
Initialization	11.34 ns	5.84 ns
Precision	4.1 ns	1.91 ns

**Table 3.** Comparison results between both analog and proposed VLSI implementations.

**Parameter**	**Analog Implementation**	**Proposed VLSI Implementation**
Power consumption	24 mW (normal)	7 mW (normal)
	22 mW (attack)	6 mW (attack)
Timing resolution	9 ns–22 ns	9 ns–10 ns
T*test*−T*clk_out*	4.39 ns	2.10 ns
Area occupied	1,306 μm^2^ + passive elements + sources	684^2^

## References

[1] Rankl W. (2003). Overview about attacks on smart cards. Inf. Security Tech. Rep..

[2] Grand J. Practical Secure Hardware Design for Embedded Systems.

[3] Weingart S.H. Physical Security Devices for Computer Subsystems: A Survey of Attacks and Defenses.

[4] McLoughlin I. Secure Embedded Systems: The Threat of Reverse Engineering.

[5] Subramanyan P., Tsiskaridze N., Pasricha K., Reisman D., Susnea A., Malik S. Reverse Engineering Digital Circuits Using Functional Analysis.

[6] Kömmerling O., Kuhn M.G. Design Principles for Tamper-resistant Smartcard Processors.

[7] Handschuh H., Paillier P., Stern J., Koç C.K., Paar C. (1999). Probing Attacks on Tamper-resistant Devices. Cryptographic Hardware and Embedded Systems.

[8] Berkes J. (2006). Hardware Attacks on Cryptographic Devices.

[9] Anderson R., Kuhn M. (1998). Low Cost Attacks on Tamper Resistant Devices. Security Protocols..

[10] Bao F., Deng R.H., Han Y., Jeng A., Narasimhalu A.D., Ngair T. (1998). Breaking Public Key Cryptosystems on Tamper Resistant Devices in the Presence of Transient Faults. Security Protocols.

[11] Ganguly A., Ahmed M.Y., Vidapalapati A. A Denial-of-Service Resilient Wireless NoC Architecture.

[12] Dhem J.F., Feyt N. (2001). Present and Future Smart Cards. Gemplus-Card Security Group.

[13] Witteman M. (2002). Advances in smartcard security. Inf. Secur. Bull..

[14] Staszewski R.B., Hung C.M., Barton N., Lee M.C., Leipold D. (2005). A digitally controlled oscillator in a 90 nm digital CMOS process for mobile phones. IEEE J. Solid State Circuits.

[15] Yu C.Y., Yu J.Y., Lee C.Y. (2012). A low voltage all-digital on-chip oscillator using relative reference modeling. IEEE Trans. Large Scale Integr. (VLSI) Syst..

[16] Kumar M., Arya S.K., Pandey S. (2012). Digital controlled oscillator design with novel 3 transistors XOR gate. Int. J. Smart Home.

[17] Hauck S. (1995). Asynchronous Design Methodologies: An Overview. Proc. IEEE.

[18] Matsui M., Hara H., Uetani Y., Lee-Sup Kim, Nagamatsu T., Watanabe Y., Sakurai T., Chiba A., Matsuda K. (1994). A 200 MHz 13 mm 2-D DCT macrocell using senseamplifying pipeline flip-flop scheme. IEEE J. Solid State Circuits.

[19] Jiménez R., Parra P., SanMartín P., Acosta A.J. (2002). Analysis of high-performance flip-flops for submicron mixed-signal applications. Analog Integr. Circuits Signal Process..

[20] Jiménez R., Feria G., Sánchez-Raya M., Galan J., Gómez F. FPGA Implementation of Hardware Countermeasures.

[21] Stevenson J.M., Sanchez-Sinencio E. (1998). An accurate quality factor tuning scheme for iF and high-Q continuous-time filters. IEEE J. Solid-State Circuits.

[22] Krummenacher F., Joehl N. (1998). A 4 MHz CMOS continuous-time filter with on-chip automatic tuning. IEEE J. Solid State Circuits.

[23] Bar-el H., Choukri H., Naccache D., Tunstall M., Whelan C. (2006). The Sorcerer's Apprentice Guide to Fault Attacks. Proc. IEEE.

